# Elevated osteoprotegerin is associated with abnormal ankle brachial indices in patients infected with HIV: a cross-sectional study

**DOI:** 10.1186/1758-2652-13-12

**Published:** 2010-03-22

**Authors:** James J Jang, Aron I Schwarcz, Daniel A Amaez, Mark Woodward, Jeffrey W Olin, Marla J Keller, Alison D Schecter

**Affiliations:** 1 Zena and Michael A Wiener Cardiovascular Institute and Marie-Joseìe and Henry R Kravis Center for Cardiovascular Health, Mount Sinai School of Medicine, New York, New York, USA; 2 Division of General Medicine, Mount Sinai School of Medicine, New York, New York, USA; 3 Division of Infectious Diseases, Mount Sinai School of Medicine, New York, New York, USA

## Abstract

**Background:**

Patients infected with HIV have an increased risk for accelerated atherosclerosis. Elevated levels of osteoprotegerin, an inflammatory cytokine receptor, have been associated with a high incidence of cardiovascular disease (including peripheral arterial disease, or PAD), acute coronary syndrome, and cardiovascular mortality. The objective of this study was to determine whether PAD is prevalent in an HIV-infected population, and to identify an association with HIV-specific and traditional cardiovascular risk factors, as well as levels of osteoprotegerin.

**Methods:**

One hundred and two patients infected with HIV were recruited in a cross-sectional study. To identify the prevalence of PAD, ankle-brachial indices (ABIs) were measured. Four standard ABI categories were utilized: ≤ 0.90 (definite PAD); 0.91-0.99 (borderline); 1.00-1.30 (normal); and >1.30 (high). Medical history and laboratory measurements were obtained to determine possible risk factors associated with PAD in HIV-infected patients.

**Results:**

The prevalence of PAD (ABI ≤ 0.90) in a young HIV-infected population (mean age: 48 years) was 11%. Traditional cardiovascular risk factors, including advanced age and previous cardiovascular history, as well as elevated C-reactive protein levels, were associated with PAD. Compared with patients with normal ABIs, patients with high ABIs had significantly elevated levels of osteoprotegerin [1428.9 (713.1) pg/ml vs. 3088.6 (3565.9) pg/ml, respectively, p = 0.03].

**Conclusions:**

There is a high prevalence of PAD in young HIV-infected patients. A number of traditional cardiovascular risk factors and increased osteoprotegerin concentrations are associated with abnormal ABIs. Thus, early screening and aggressive medical management for PAD may be warranted in HIV-infected patients.

## Background

HIV infection is an epidemic affecting an estimated 33 million people worldwide, with approximately 40,000 new cases reported each year in the United States [1]. There is evidence of accelerated atherosclerosis among young patients infected with HIV [2]. Three recent epidemiologic studies have reported an increased prevalence of peripheral arterial disease (PAD) in HIV-infected patients [3-5]. However, there is a paucity of clinical data on the predictive risk factors and biologic markers associated with PAD in HIV-infected patients.

Potential hypotheses for accelerated atherosclerosis in HIV-infected patients include metabolic derangements and direct effects of protease inhibitors (PIs), as well as a primary impact of the HIV infection resulting in vasculopathy and vascular inflammation [2,6-8]. Recently, PI use was found to be associated with PAD in HIV-infected patients [9].

Peripheral arterial disease affects approximately 8 to 12 million people in the US and is an eminently treatable disease [10]. Individuals with PAD have a seven- to 10-fold increased risk of cardiovascular ischemic events and a short-term mortality that is increased at least three fold compared with individuals without PAD at a similar age [11]. The diagnosis of PAD has traditionally been identified by detecting an ankle-brachial index (ABI) equal to or less than 0.90. Recently, it has been demonstrated that low ABIs (<1.10) and elevated ABIs (>1.40), which were previously considered normal, are associated with an increase in all-cause and cardiovascular mortality [12].

Atherosclerosis has been well described as an inflammatory process [13]. Osteoprotegerin (OPG), a member of the tumor necrosis factor receptor family, inhibits receptor activator of nuclear factor-κB ligand (RANKL) [14]. OPG has been implicated in bone remodelling, as well as in atherosclerotic progression, vascular calcification and vascular inflammation [15-18]. Moreover, elevated levels of OPG have correlated with the onset of cardiovascular events and an increased severity of PAD [19,20].

The objective of this study was to evaluate the prevalence of, and risk factors associated with PAD in an urban, HIV-infected population. The identification and validation of non-invasive and surrogate markers of vascular inflammation, such as OPG, in HIV-infected patients may have a beneficial impact on early detection and treatment for those with PAD.

## Methods

### Study population

Men and women 18 years of age or older with documented HIV infection were recruited from the Jack Martin Fund Clinic, a New York State designated AIDS center, located at the Mount Sinai Medical Center in New York, New York. The clinic provides primary and subspecialty care to approximately 1800 HIV-infected patients. The ethnic make up of the clinic reflects a heterogeneous population within urban New York: 45% are women, 45% are Hispanic, 44% are African American, and less than 1% are Asian. Inclusion criteria included HIV infection as documented by enzyme immunoassay and confirmed by western blot analysis or a detectable plasma HIV-1 RNA at any time prior to study entry.

This study was approved by the Institutional Review Board of the Mount Sinai School of Medicine. All participants provided written informed consent. Since this study was intended to identify PAD secondary to atherosclerosis, exclusion criteria included the diagnosis of vascular disease of non-atherosclerotic origin, such as vasculitis (i.e. giant cell arteritis, Takayasu's disease, Buerger's disease). There was no exclusion based on gender, socio-economic, racial or ethnic backgrounds.

### Study design

This was a cross-sectional study. Subjects were recruited from December 2005 to May 2006 by investigators with the intention of enrolling patients on consecutive clinic days to include patients cared for by all clinic providers. This recruitment strategy was adopted to limit selection bias. After informed consent was obtained, patients were interviewed for demographic information, including gender, ethnicity and birth date.

A limited physical exam was then performed to measure blood pressure (BP), pulse, height, weight and waist circumference. Body mass index (BMI) was calculated by dividing the weight in kilograms by the square of the height in meters. In addition, medical charts were obtained and reviewed for each patient recruited into the study.

### Cardiovascular risk factor evaluation

The presence of diabetes mellitus was determined by self-report, chart documentation, or the use of diabetic medication. The diagnosis of dyslipidemia was determined by self-report, clinical record, or the use of lipid-lowering agents. In addition, patients were identified as dyslipidemic if any of the lipid profiles from their medical record met National Cholesterol Education Panel (NCEP) criteria [21]. Fasting glucose or lipid profiles were not obtained as part of the protocol. Hypertension was defined by self-report, chart documentation, or use of anti-hypertensive medications.

A diagnosis of metabolic syndrome was determined by identifying three of the following five criteria, as defined by the NCEP: central obesity as measured by waist circumference; high BP; glucose intolerance; high triglyceride levels; and low high density lipoprotein (HDL) cholesterol concentration [21]. Patients were questioned on previous and current smoking use.

A positive family history was defined as any first-degree relative with a history of cardiovascular events in a male under 55 years and female under 65 years. History of cardiovascular and cerebrovascular diseases was obtained by self-report and/or chart review. History of cardiovascular disease, including documented history of coronary artery disease, was based on a history of stable or unstable angina, myocardial infarction, percutaneous coronary intervention, or coronary artery bypass graft surgery. History of cerebrovascular disease was defined as a history of transient ischemic attack, ischemic or hemorrhagic stroke. The diagnosis of PAD was based on a history of abnormal ABIs, percutaneous peripheral arterial intervention, and peripheral arterial bypass surgery.

### HIV risk evaluation

Patients were interviewed regarding their previous and current HIV medical history. Based on this interview and medical chart review, the duration of HIV infection was determined. The duration of protease inhibitor use was obtained and recorded as total months. Current CD4 count and viral load were determined by reviewing the most recent laboratory results.

### Ankle-brachial index measurements

The ankle-brachial index was measured by three study participants (JJJ, AIS, DAA) who were trained by the accredited vascular diagnostic laboratory at the Mount Sinai Medical Center according to standardized laboratory procedures. Patients were placed in a supine position following a five-minute rest period. While the patient was supine, a BP cuff (Tycos, Welch Allyn, Skaneateles Falls, NY) was placed just above the elbow. An 8 mHz continuous wave hand-held Doppler transducer probe (Nicolet Vascular, Madison, WI) was positioned over the brachial artery. The BP cuff was then inflated until the pulse signal was obliterated and inflation continued another 20 mmHg. After slowly releasing the cuff pressure, the first audible tone was recorded as the brachial systolic BP. This was repeated for both arms and the highest brachial pressure was used for the ABI calculation.

After both brachial artery blood pressures were obtained, the BP cuff was placed approximately five centimeters above the medial malleolus on each lower extremity. The Doppler probe was positioned over the posterior tibial (PT) arteries. The BP cuff was then inflated until the pulse signal was obliterated and inflation continued another 20 mmHg. After slowly releasing the cuff pressure, the first audible tone was recorded as the ankle systolic BP. This procedure was then repeated on the opposite ankle for the PT systolic pressures, as well as both arms above the elbow for brachial systolic pressures.

For this study, only PT pressures were used to determine ABIs. The PT-only ABI method was chosen since numerous large PAD epidemiological studies, including National Health and Nutrition Examination Survey (NHANES), used this technique [22-26]. The dorsalis pedis pressure was used when the PT systolic pressure was inaudible. The recorded ankle pressure was divided by the highest brachial artery systolic pressures of either arm.

The lower ABI of either limb was used to categorize the patients into four designated ABI categories. The ABI categories defined in this study include definite PAD (ABI ≤ 0.90), borderline ABI (ABI = 0.91-0.99), normal ABI (ABI = 1.00-1.30), and high ABI (ABI >1.30). The four ABI categories used in this study were similar to those previously described to not only diagnose PAD, but also to identify patients that may be at increased risk for cardiovascular events [27].

### Blood analysis

Complete blood count, basic chemistry panel and lipid profiles were recorded from the patient's most recent laboratory test results. Plasma samples were analyzed for inflammatory markers, including OPG, C-reactive protein (CRP), interleukin-1β (IL-1β), and interleukin-6 (IL-6). The IMMAGE 800 assay (Beckman Coulter, Fullerton, CA, USA), using a polyclonal anti-C-reactive protein antibody coated to latex particles, was used to measure CRP concentrations. The IMMAGE CRPH is based on the highly sensitive Near Infrared Particle Immunoassay rate methodology (Beckman Coulter, Fullerton, CA, USA). IL-1β, IL-6, and OPG were all assayed using a quantitative sandwich immunoassay technique (R&D Systems, Inc., Minneapolis, MN, USA). Antibodies to IL-1β, IL-6 were *E. coli*-derived and antibodies to OPG were derived from a murine myeloma cell line (R&D Systems, Inc., Minneapolis, MN, USA).

### Statistical analysis

Associations between continuous variables and ABI were tested using general linear models, after first transforming to approximate normality, where necessary. Logarithmic transformations were used for: glucose and OPG; square root transformations for CD4, and both PI and HIV durations; and reciprocal transformations for viral load. Associations between binary variables and ABI were tested using logistic regression models. All models included contrasts to obtain statistics that compare each other group to normal ABI (the reference group). All associations were tested before and after adjustment for potential confounding factors: age, sex, BMI, smoking, diabetes mellitus, total cholesterol, HDL, low density lipoprotein, triglycerides, CRP, cardiovascular disease, family cardiac history, duration of HIV and duration of PI use. For all analyses, a p value < 0.05 was considered statistically significant.

## Results

### Prevalence of PAD

The average age of the study population was 48.4 years old. The prevalence of PAD (ABI ≤ 0.90) in this relatively young HIV-infected population was 11%. Only 56% of the cohort had ABI measurements that were considered normal (ABI 1.00-1.30). Of the remaining study population, 18% had borderline ABIs (0.91-0.99), while 15% had high ABIs (ABI >1.30) (Table S1, Additional file 1).

### Risk factors for PAD associated with HIV infection

Potential HIV-specific risk factors, including duration of protease inhibitor use, HIV exposure duration, CD4 count and viral load, were evaluated. However, none of these risk factors were found to be independently predictive of abnormal ABIs in this cohort.

### Cardiovascular risk factors associated with PAD

Despite the high prevalence of PAD identified by this study, the majority of patients did not have traditional cardiovascular risk factors as defined by the Framingham risk criteria: dyslipidemia (23%), hypertension (28%), diabetes (12%), family cardiac history (23%), and metabolic syndrome (25%) [28]. However, advanced age significantly correlated with definite PAD compared to normal ABIs [mean: 54.2 (12.8) years vs. 47.3 (8.0) years, respectively; p = 0.02]. In addition, previously documented cardiovascular disease was significantly associated with PAD (p = 0.0005). Although 75% of the cohort had a smoking history, smoking was not an independent risk factor for PAD in this study.

### Biomarkers for PAD

To assess for an association between inflammatory biomarkers for PAD in HIV-infected participants, CRP, IL-1β, IL-6, and OPG levels were measured. Elevated CRP levels were significantly associated with definite PAD. Concentrations of OPG were significantly elevated in patients with high ABIs compared with patients with normal ABIs [mean: 3088.6 (3565.9) pg/ml vs. 1428.9 (713.1) pg/ml, respectively; p = 0.03]. Levels of IL-1β, and IL-6 did not significantly differ across ABI groups (Table S1, Additional file 1).

## Discussion

The salient observations from this study are that in this relatively young, urban, HIV-infected cohort (1) there is an 11% prevalence of PAD; (2) many HIV-infected individuals have abnormal ABIs, a known marker of increased risk for cardiovascular events and mortality; and (3) elevated OPG levels are associated with high ABIs.

Based on large cross-sectional studies that used the same ABI technique as in our study, the prevalence of PAD (defined as ABI <0.90) was 12.4% in the Cardiovascular Health Study, 19.1% in the Rotterdam Study, 18.0% in the Edinburgh Study, and 3.0% in the Atherosclerosis Risk in Communities study [22-25]. Interestingly, the mean age of the aforementioned studies was 71.7-75.7 years, 69.0-71.7 years, 65.6-67.7 years, and 53.0-55.0 years, respectively [22-25]. The mean age of the present study cohort was 48.4 years. Despite being a significantly younger mean age, our cohort had an 11% prevalence of PAD. In the National Health and Nutrition Examination Survey (NHANES), the prevalence of PAD in patients aged 40 to 49 years was only 0.6-1.1% [26]. Thus, HIV-infected patients at similar ages to our cohort may have an increased risk of PAD compared with patients without HIV.

In addition to identifying patients with definite PAD (ABI ≤ 0.90), the remainder of the cohort were classified into three other ABI categories, defined as borderline (ABI = 0.91-0.99), normal (ABI = 1.00-1.30) and high (ABI >1.30). It has been well documented that patients with ABIs <0.90 are two times more likely to have cardiovascular events than patients with normal ABIs [25,29]. However, borderline ABIs (0.91-0.99), that previously were considered normal, have now been associated with mortality or cardiovascular disease morbidity of approximately 15% at six years [22]. Based on the Strong Heart Study, patients with borderline ABIs (0.90-0.99, n = 195) had approximately 30% increased risk for all-cause mortality and approximately 10% increased risk for cardiovascular mortality [12]. In our HIV-infected cohort, the prevalence of patients with ABIs = 0.91-0.99 was 18%. This is especially important given that by Framingham risk criteria, the majority of the patients in this study would be classified as low risk (<10%) for cardiovascular events and therefore would not have been screened according to current American College of Cardiology/American Heart Association (ACC/AHA) PAD practice guidelines [28,30].

Recently, elevated ABIs, that previously were considered normal, have been associated with a significant risk for cardiovascular mortality [12]. The Multi-Ethnic Study of Atherosclerosis (MESA) found that men with ABIs ≥ 1.30 had significantly elevated coronary calcium scores compared with men with normal ABIs [27]. Interestingly, the mean age of the MESA cohort was 63.4 years, yet the prevalence of ABIs ≥ 1.30 was only 5.7% [27].

In the present cohort, with a mean age of 48.4 years, the prevalence of ABIs >1.30 was 15%. Recently, Sharma *et al *reported the prevalence of elevated ABIs in HIV-infected women to be 7.2% [3]. Similarly, the prevalence of elevated ABIs in our cohort of HIV-infected women was 5%. In contrast, 10% of the HIV-infected men had elevated ABIs. By combining all of our patients with low, borderline and high ABI results, approximately 44% of our cohort had ABIs that put them at significant risk for cardiovascular events and mortality.

PAD is strongly associated with traditional cardiovascular risk factors, such as advanced age, gender, dyslipidemia, hypertension, diabetes and tobacco use [31]. In this study, advanced age and previously documented cardiovascular disease (i.e., coronary artery disease, myocardial infarction and stroke) was significantly associated with definite PAD. From the NHANES database, there is almost a doubling in the prevalence of PAD in men with each decade of life from 40 to 70 years [26].

The oldest subgroup in the present study had a mean age of 54.2 years. Despite being the oldest subgroup in this study, they are considerably younger than previously studied cohorts [22-25]. The NHANES study also reported that approximately 33% of patients with PAD had previously documented cardiovascular disease [26]. In this present study, there was only a 13% incidence of previous cardiovascular disease.

Inflammatory responses appear to mediate atherogenesis [13]. In our study, we observed that elevated CRP concentrations are associated with definite PAD in our cohort. Similarly, the NHANES study found that after adjusting for traditional cardiovascular disease risk factors, patients with highest quartile of CRP had a 2.1-fold increased odds for PAD [32].

Osteoprotegerin, a member of the tumor necrosis factor receptor family, inhibits receptor activator of nuclear factor-κB ligand (RANKL) [14]. OPG has been identified as a regulator of bone formation and resorption [15]. OPG is found not only in bone, but also in the blood vasculature (endothelium and smooth muscle cells) where it plays a role promoting advanced atherosclerosis, calcification, and inflammation [16-18]. Elevated levels of OPG have been associated with an increased incidence of cardiovascular disease (including PAD), acute coronary syndrome, and cardiovascular mortality [19,33].

Although inflammatory markers, such as CRP, IL-1β, and IL-6, are associated with cardiovascular diseases, OPG is a unique biomarker in that elevated levels have independently correlated with progression of coronary artery calcification [34]. From our HIV-infected cohort, elevated OPG levels, rather than CRP, IL-1β, and IL-6, were found to be associated with high ABIs. This is the first study to document a correlation between elevated OPG levels with high ABIs in either HIV- or non-HIV-infected patients. Interestingly, a number of previous studies have observed that HIV-infected patients have increased OPG levels compared to matched, non-HIV-infected patients [35,36].

A few important limitations of this study deserve consideration. The sample size is relatively small compared with other prevalence studies evaluating PAD in HIV-uninfected individuals. It is possible that certain cardiovascular and HIV risk factors may have reached or failed to reach statistical significance as predictors for PAD due to the small sample size of our study. Also, we did not include a control group of HIV-uninfected patients to serve as a comparison group. We cannot infer on the mortality risk of our cohort with abnormal ABIs based on data from previous studies of HIV-uninfected patients. Perhaps, a future study investigating the risk of mortality in HIV-infected patients with abnormal ABIs may be warranted.

## Conclusions

In summary, HIV-infected patients have a high prevalence of PAD. Many patients with HIV have abnormal ABIs, thus placing them at an increased risk for cardiovascular events and mortality. A number of cardiovascular risk factors, as well as elevated concentrations of OPG, correlated with abnormal ABIs in HIV-infected patients. Given the high prevalence and significant clinical consequences associated with abnormal ABIs and elevated OPG levels, early cardiovascular screening and aggressive medical management may be warranted in HIV-infected patients.

## Competing interests

The authors declare that they have no competing interests.

## Authors' contributions

JJJ was responsible for study concept and design, data analysis, interpretation of the study findings, and manuscript writing. AIS and DAA assisted in collecting data and creating the database, the interpretation of study findings, and the critical revision the manuscript. MW assisted in data and statistical analysis, interpretation of study findings, and the critical revision of the final manuscript. JWO, MJK and ADS assisted in the interpretation of study findings and critical revision of the manuscript. All authors read and approved the final manuscript.

## Supplementary Material

Additional file 1 **Table S1: Characteristics of 102 HIV-infected patients at the Jack Martin Fund Clinic, Mount Sinai Medical Center, New York, New York**. Data are presented as mean (standard deviation) for continuous variables and number (No., %) for binary variables. ABI = ankle-brachial index. SD = standard deviation. HDL = high density lipoprotein. LDL = low density lipoprotein. IL-1β = Interleukin-1β. IL-6 = interleukin-6. CVD = cardiovascular disease. Click here for file
