# Supplementary material for: Elevated osteoprotegerin is associated with abnormal ankle brachial indices in patients infected with HIV: a cross-sectional study
Source: J Int AIDS Soc. 2010 Mar 22;13:12. doi: 10.1186/1758-2652-13-12 (PMC2859852; doi:10.1186/1758-2652-13-12)
Supplement: Additional file 1 — Table S1: Characteristics of 102 HIV-infected patients at the Jack Martin Fund Clinic, Mount Sinai Medical Center, New York, New York. Data are presented as mean (standard deviation) for continuous variables and number (No., %) for binary variables. ABI = ankle-brachial index. SD = standard deviation. HDL = high density lipoprotein. LDL = low density lipoprotein. IL-1β = Interleukin-1β. IL-6 = interleukin-6. CVD = cardiovascular disease. [file 1758-2652-13-12-S1.DOC]

# Table S1. Summary statistics for 102 HIV-infected patients

# Characteristics Total Definite PAD Borderline ABI Normal ABI High ABI

(**ABI ≤ 0.90) (ABI 0.91-0.99) (ABI 1.00-1.30) (ABI >1.30)**

# Continuous variables: [Mean value (SD)]

Patient total (%) 102 11(11) 19 (18) 57 (56) 15 (15)

Age (years) 48.4 (9.1) 54.2 (12.8) * 49.0 (9.1) 47.3 (8.0) 47.6 (9.1)

Waist circumference (cm) 94.3 (14.2) 96.8 (17.3) 86.6 (11.2) 93.8 (13.7) 100.0 (13.8)

Body mass index (kg/m^2^) 27.4 (6.0) 28.4 (6.9) 23.6 (4.1) 27.6 (6.1) 29.9 (4.6)

White blood cell (x10^3^/uL) 5.8 (1.8) 6.1 (2.3) 5.4 (1.7) 5.7 (1.8) 6.2 (1.9)

Hemoglobin (g/dL) 13.3 (1.8) 13.2 (1.8) 13.4 (1.6) 13.1 (1.9) 13.4 (2.4)

Platelets (x10^3^/uL) 230.4 (76.9) 239.4 (90.7) 220.7 (87.2) 226.3 (70.1) 235.3 (84.4)

Glucose (mg/dL) 95.7 (44.4) 118.7 (102.0) 91.0 (21.1) 91.5 (37.1) 81.5 (9.2)

Total cholesterol (mg/dL) 166.4 (39.8) 163.1 (21.0) 157.1 (37.1) 170.3 (43.6) 174.9 (38.0)

HDL (mg/dL) 46.0 (16.2) 43.8 (17.2) 44.1 (13.1) 48.8 (17.9) 47.4 (12.4)

LDL (mg/dL) 84.1 (37.0) 77.6 (22.2) 80.2 (29.1) 85.7 (41.3) 92.7 (38.7)

Triglycerides (mg/dL) 189.2 (111.0) 209.7 (116.6) 175.8 (132.6) 190.6 (108.8) 187.7 (94.6)

Duration of HIV (months) 141.1 (63.6) 136.0 (81.0) 158.7 (67.9) 152.7 (57.7) 116.8 (62.1)

Duration of PI use (months) 52.7 (56.6) 35.3 (49.5) 37.6 (58.2) 62.6 (58.7) 47.6 (47.5)

HIV viral load (copies/mL) 10,856.5 (47682.8) 4364.0 (10748.4) 21,245.0 (81947.2) 14,440.1 (42449.6) 3376.9 (8308.7)

CD4 cell count (cells/mL) 565.4 (416.3) 428.6 (208.2) 504.1 (378.5) 601.6 (486.3) 606.1 (249.4)

C-reactive protein (mg/L) 6.5 (6.8) 8.5 (6.3)* 4.6 (4.0) 7.2 (8.5) 5.1 (4.0)

IL-1β (pg/mL) 6.6 (4.8) 8.1 (6.6) 5.2 (2.5) 6.6 (5.0) 7.1 (5.5)

IL-6 (pg/mL) 16.6 (22.8) 27.7 (45.4) 18.9 (30.0) 11.6 (9.3) 24.8 (48.6)

Osteoprotegerin (pg/mL) 1741.7 (1244.3) 2237.9 (1830.5) 1329.5 (665.8) 1428.9 (713.1) 3088.6 (3565.9)*

**Binary variables: [No. (%)]**

Male sex (%) 58 (57) 5 (45) 13 (68) 29 (51) 10 (67)

Hypertension (%) 29 (28) 5 (45) 1 (5) 19 (33) 5 (33)

Diabetes mellitus (%) 12 (12) 2 (18) 2 (11) 7 (12) 1 (7)

Dyslipidemia (%) 23 (23) 3 (27) 3 (16) 12 (21) 5 (33)

Smoking history (%) 76 (75) 8 (73) 16 (84) 43 (75) 9 (60)

Metabolic syndrome (%) 26 (25) 3 (27) 6 (32) 14 (25) 3 (20)

Family cardiac history (%) 23 (23) 1 (9) 4 (21) 12 (21) 6 (40)

CVD history (%) 13 (13) 6 (55)* 2 (11) 4 (7) 1 (7)

* p<0.05 compared to normal ABI, after adjustment for age, sex, BMI, smoking, diabetes mellitus, total cholesterol, HDL, LDL, triglycerides, CRP, CVD, family cardiac history, duration of HIV and duration of PI use (hypertension only adjusted for age and sex)
